# Chancre on the Finger of a Dentist Supposed to Be Communicated from the Mouth of a Patient

**Published:** 1858-10

**Authors:** 


*Chancre on the finger of a Dentist supposed to be communicated from the mouth of a Patient.*—We copy the following case from the *Boston* Medical and Surgical Journal, as an important caution. The history is necessarily imperfect, as the operator evidently had no suspicion of any of his patients until the progress of the disease disclosed the manner in which he had been poisoned.

Chancres are by no means uncommon in the mouth; indeed they are found upon nearly all the exposed mucous surfaces. Their peculiar poison cannot be communicated except through a broken or abraded cuticle, but this is common enough upon the most carefully tended hands. In the general practice of medicine the accident here described is by no means unknown. Physicians, however, being subject to greater risks in this respect are more cautious, and indeed have usually sufficient warning to put them upon their guard. It would be well for dentists to exercise the same salutary caution, and to scrutinize closely all suspicious sores in the mouths of their patients. They may possibly find chancres in mouths where they would least suspect them.

EDS.

About the 20th August, 1857, I noticed just above the nail, on the middle finger of the left hand, an oblong, red spot, about the size of a three cent piece. To protect it, I covered it with court plaster. In about a week a vesicle formed, which soon broke, discharged slightly, and was followed by an ulcer about the size of the original red spot. About September 3d, I consulted Dr. A. It was then an indolent ulcer, giving no pain, and he thought it was perhaps derived from a foul tooth in operating. *Treatment*, application of nitrate of silver, and compression.

Sept. 8th, ulcer rather worse. There was swelling of a gland in the upper part of the arm, but below the axilla. Consulted Dr. B. The ulcer was then too much irritated to enable him to decide as to its character. *Treatment*, poulticing.

Sept. 12th, another gland just above the elbow became inflamed. The skin over both glands was red and hot, motion of the elbow impeded, ulcer a little extended on one side, and down to the nail. Continue poultices, and take Blancard's pills (iodide of iron) three times a day.

Sept. 26th, Dr. B. being out of town, I consulted Dr. C., who called on Dr. D. to look at it. Dr. D. said it looked like a chancre. Both agreed in fear that it was malignant. They advised black wash, and doubling the dose of the iodide. Being troubled by this, I consulted Dr. E., who said it was not malignant, but probably like a dissecting-room sore. He advised black wash, and attention to general health.

Sept. 30th, I visited Dr. B. again. He was much dissatisfied with the appearance of the sore, and cauterized it with the acid nitrate of mercury. The application produced extreme pain for about two hours, and at intervals for some hours longer. A dry eschar was produced, from beneath which escaped, on the third or fourth day, a drop of pus. This was the first genuine pus which the sore had produced.

Oct. 14th, eschar separating at the edge. Dr. B. advised a journey and exercise in the open air, as the general health was now much affected.

Oct. 21st, finger somewhat irritable. Eschar loosening. Visited Dr. F., who said that the finger was poisoned, probably by syphilis, as a papular eruption was just appearing. He recommended corrosive sublimate in doses of one-sixteenth of a grain, three times a day, combined with tonics, and good, but plain diet. He sent me to Dr. G., who coincided with him. 23d, eruption fully developed. *Diagnosis.* Indurated chancre on the finger, papular eruption, of fully marked syphilitic character. It is useless to pursue the case farther, except to say that the progress has fully verified the diagnosis.

My object in giving this account, and in this form, is to call the attention of dentists (and their physicians) to the fact that in the practice of their art they may meet with a similar misfortune, and that its character may not easily be perceived by the most skillful surgeons. In New York, I was assured by Dr. F. (who is a professor, and a very distinguished surgeon,) that chancres in the mouth are by no means rare, and perhaps a search would prove them about as plenty in Boston. Should the finger come in contact with such a sore, a hang-nail would give abundant entrance to the poison.

